# Testis-expressed gene 11 inhibits cisplatin-induced DNA damage and contributes to chemoresistance in testicular germ cell tumor

**DOI:** 10.1038/s41598-022-21856-3

**Published:** 2022-11-01

**Authors:** Sachi Kitayama, Kazuhiro Ikeda, Wataru Sato, Hideki Takeshita, Satoru Kawakami, Satoshi Inoue, Kuniko Horie

**Affiliations:** 1 Division of Systems Medicine and Gene Therapy, Saitama Medical University, Hidaka, Saitama 350-1241 Japan; 2 Department of Urology, Saitama Medical Center, Saitama Medical University, Kawagoe, Saitama 350-8550 Japan; 3 Department of Systems Aging Science and Medicine, Tokyo Metropolitan Institute of Gerontology, Itabashi-ku, 35-2 Sakae-cho, Tokyo, 173-0015 Japan

**Keywords:** Cancer, Cell biology, Molecular biology

## Abstract

Testicular germ cell tumor (TGCT) is a rare cancer but the most common tumor among adolescent and young adult males. Patients with advanced TGCT often exhibit a worse prognosis due to the acquisition of therapeutic resistance. Cisplatin-based chemotherapy is a standard treatment for advanced TGCTs initially sensitive to cisplatin, as exemplified by embryonal carcinoma. The acquisition of cisplatin resistance, however, could be a fatal obstacle for TGCT management. To identify cisplatin resistance-related genes, we performed transcriptome analysis for cisplatin-resistant TGCT cells compared to parental cells. In two types of cisplatin-resistant TGCT cell models that we established from patient-derived TGCT cells, and from the NEC8 cell line, we found that mRNA levels of the high-mobility-group nucleosome-binding gene *HMGN5* and meiosis-related gene *TEX11* were remarkably upregulated compared to those in the corresponding parental cells. We showed that either HMGN5 or TEX11 knockdown substantially reduced the viability of cisplatin-resistant TGCT cells in the presence of cisplatin. Notably, TEX11 silencing in cisplatin-resistant TGCT cells increased the level of cleaved PARP1 protein, and the percentage of double-strand break marker γH2AX-positive cells. We further demonstrated the therapeutic efficiency of *TEX11*-specific siRNA on in vivo xenograft models derived from cisplatin-resistant patient-derived TGCT cells. Taken together, the present study provides a potential insight into a mechanism of cisplatin resistance via TEX11-dependent pathways that inhibit apoptosis and DNA damage. We expect that our findings can be applied to the improvement of cisplatin-based chemotherapy for TGCT, particularly for TEX11-overexpressing tumor.

## Introduction

Testicular germ cell tumor (TGCT) is a rare cancer but the most common malignancy among adolescent and young adult males between 14 and 44 years of age, and its incidence is increasing worldwide^1^. The application of cisplatin-based chemotherapy combined with surgery has remarkably improved the prognoses of TGCT patients, particularly in developed countries, although some patients suffer from chemoresistance that results in a late relapse^2^. The elucidation of molecular mechanisms underlying cisplatin sensitivity and resistance will help us to understand more precisely the pathophysiology of advanced TGCT, and to develop alternative diagnostic and therapeutic options for refractory diseases.

Cisplatin resistance in tumors cells have been categorized into several molecular mechanisms based on the action points, including pre-target, on-target, post-target, and off-target responses^3^. In terms of on-target actions, intrastrand and interstrand crosslinks are elicited by cisplatin as DNA lesions, which are recognized by several pathways for DNA damage responses^4^. Cisplatin-dependent intrastrand adducts are basically removed by nucleotide excision repair. In the process, high mobility group (HMG) proteins are known to participate in DNA repair induced by intrastand adducts^5^.

Cisplatin-based interstrand crosslinks (ICLs) can elicit DNA double-strand breaks (DSBs) because of the obstruction of DNA replication fork progression in dividing cells, and the replication-associated DSBs are often repaired by homologous recombination (HR) machinery^3^. The phosphorylated form of the histone protein H2AX, γH2AX, is a checkpoint protein for the HR DNA repair system and the sustained signal of γH2AX may indicate a defective HR system in cancer cells treated with cisplatin^6^. Poly (ADP-ribose) polymerase 1 (PARP1) is a sensor for DNA strand breaks, which regulates single-strand breaks with low-levels of DNA damage, whereas it induces γH2AX and the recruitment of BRCA1/2 to repair DSBs with high-levels of DNA damage^7^. Notably, the inability to repair ICLs is a critical feature of Fanconi Anemia (FA), a genetic disorder with bone marrow failure and cancer predisposition, thus the cells deficient in the FA pathways including BRCA1/2 usually exhibit hypersensitivity to cisplatin^8^. In the cisplatin-related DNA damage repair mechanisms, the expression of ERCC1-XPF endonuclease complex is critical for the repair of both intrastrand and interstrand DNA-cisplatin adducts, and the reduced expression of these proteins have been shown as a reason for cisplatin hypersensitivity in TGCT cells^9,10^.

In normal testicular development, primordial germ cells (PGCs) differentiate into self-renewing stem cells and meiosis of the testicular stem cells occurs postnatally. While gene mutations often occur during spermatogenesis and meiosis, mutations also take place in the early PGC population^11^. The ICL repair pathway including ERCC1 is important for normal spermatogenesis, because its deficiency leads to genomic instability and infertility^12^. In the first stage of meiosis, synapsis of homologous chromosomes occurs by the induction of programmed SPO11-induced DSBs^13–15^. Intriguingly, cisplatin is shown to increase meiotic crossing-over^16^. In SPO11-lacking meiocytes, cisplatin treatment exhibits the improvement of meiotic synapsis, suggesting the exogenous DSBs may substitute for the programmed meiotic DSBs in the synapsis^15,17^. While TGCTs are not normal descendants of PGCs or gonocytes, TGCTs including both seminoma and various types of nonseminomas originate from a common precursor germ cell neoplasia in situ (GCNIS) according to the World Health Organization classification 2016^18^. Considering that GCNIS shares many features with PGCs or gonocytes such as expression of OCT3/4 and SOX17^19,20^, we speculate whether meiotic synapsis-related factors may contribute to the DNA repair system for cisplatin-dependent DSBs and chemoresistance in TGCT.

Recently, we established a patient-derived TGCT model from embryonal carcinoma (EC)^21^, a pluripotent stem cell-like nonseminoma expressing CD30 and SOX2 as useful immunohistochemical markers^22^. While cisplatin is initially effective for the treatment of TGCT including EC, cisplatin sensitivity in EC cell lines is dependent on the extent of reduced proficiency in HR in addition to a deficiency in the ERCC1/XPF-dependent repair system^23^. To further elucidate the molecular mechanisms underlying cisplatin resistance in EC models, we generated cisplatin-resistant EC cell cultures from patient-derived TGCT cells as well as from the NEC8 EC cell line, and identified TEX11 as a novel molecular target that may be involved in cisplatin resistance, which then can be applied to clinical management for advanced TGCT.

## Results

### Generation of cisplatin-resistant TGCT cells and evaluation of their cisplatin sensitivity

**Figure 1:**
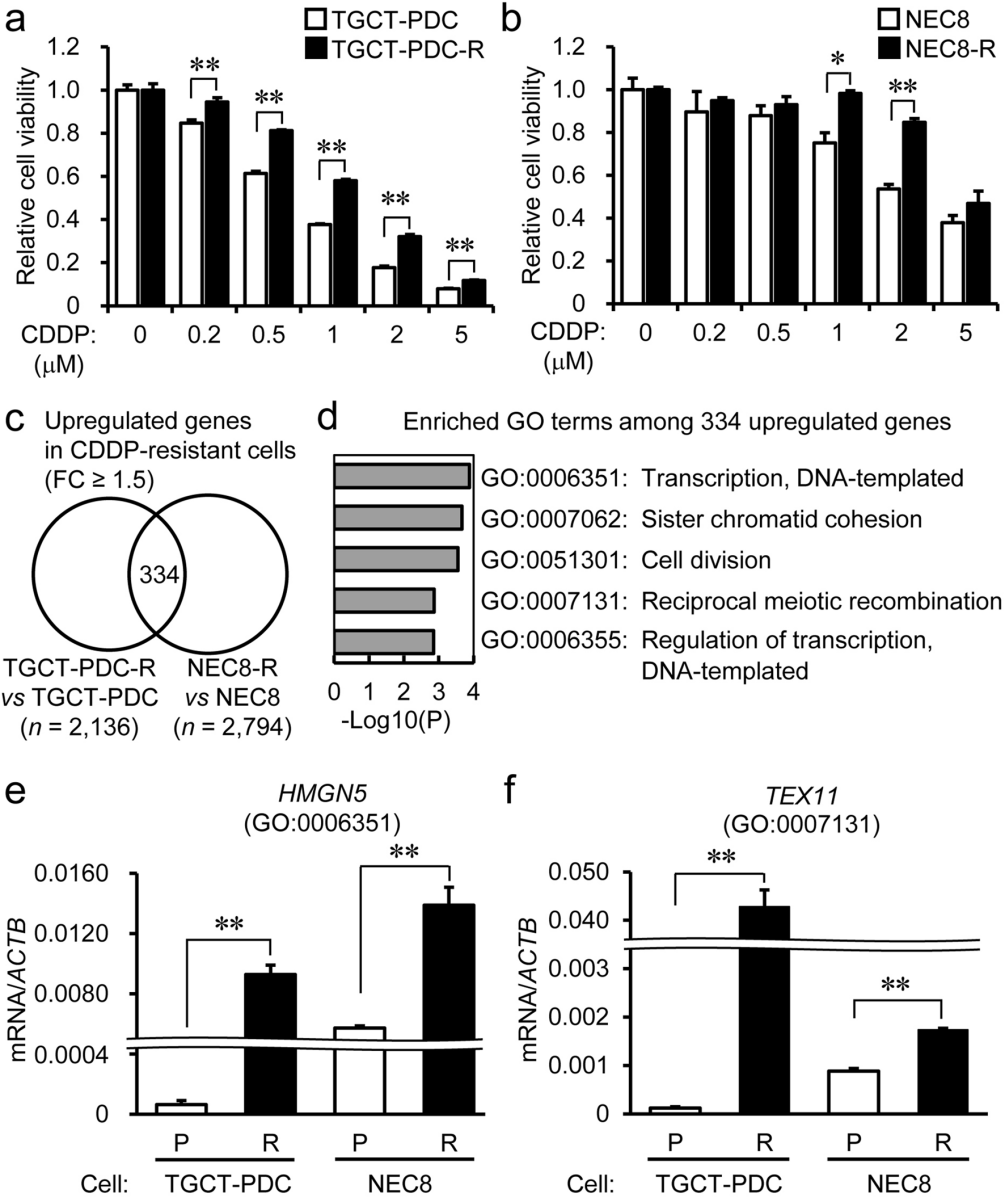
Generation of cisplatin-resistant testicular germ cell tumor (TGCT) cells and identification of upregulated genes in cisplatin-resistant TGCT cells versus parental cells. **(a)** Inhibitory effects of cisplatin to the viability of parental and cisplatin-resistant TGCT patient-derived cells (TGCT-PDC). Cells were treated with cisplatin at indicated concentrations for 96 h and subjected to the quantitation of intracellular ATP content. CDDP, cisplatin. TGCT-PDC-R, cisplatin-resistant TGCT-PDC. Results are shown as mean fold change ± SE of relative luciferase activity (n = 3). **(b)** Inhibitory effects of cisplatin to the viability of parental and cisplatin-resistant NEC8 cells. Cells were treated with cisplatin at indicated concentrations for 72 h and subjected to WST-8 cell proliferation assay. Results are shown as mean fold change ± SE of relative absorbance at 450 nm wavelength (n = 5). NEC-R, cisplatin-resistant NEC8 cells. **(c)** Microarray analysis identified 334 overlapping genes commonly upregulated by ≥ 1.5 fold with a fluorescence signal ≥ 5 in cisplatin-resistant cells compared to the corresponding parental cells. **(d)** Top 5 signaling pathways enriched among the 334 commonly upregulated genes in cisplatin-resistant TGCT-PDC and NEC8 cells based on Gene Ontology Term. **(e,f)** Overexpression of *HMGN5* **(e)** and *TEX11* **(f)** in cisplatin-resistant TGCT cells. *P*, parental cells. R, cisplatin-resistant cells. Results are shown as mean ± SE (n = 3). **, *P* < 0.01.

We experimentally generated cisplatin-resistant TGCT cells from patient-derived TGCT-PDC and the NEC8 EC cell line, designated as TGCT-PDC-R and NEC8-R cells, respectively, by a > 4-month exposure to cisplatin with stepwise increase in concentrations. The viability of TGCT-PDC-R cells was significantly higher than that of the parental cells in media including cisplatin at 0.2 µM or higher concentrations (Fig. 1a), whereas the viability of NEC8-R cells was substantially higher than that of the parental cells in media including cisplatin at 1 or 2 µM (Fig. 1b).

### Identification of upregulated genes in cisplatin‐resistant TGCT cells versus their corresponding parental cells

To characterize the difference of gene expression profiles between parental and cisplatin-resistant TGCT cells, we performed expression microarray analysis for TGCT-PDC-R versus TGCT cells and for NEC8-R versus NEC8 cells. The experiments identified 334 and 472 genes commonly upregulated and downregulated by ≥ 1.5‐ and 0.67-fold changes, respectively, in cisplatin-resistant TGCT-PDC-R and NEC8-R cells (with fluorescence signals ≥ 5 in cisplatin-resistant and parental cells, respectively) compared to their corresponding parental cells (Fig. 1c, Supplementary Fig. 1a, and Supplementary Tables 1, 2). Pathway analysis based on Gene Ontology (GO) showed that the GO term “transcription, DNA-templated” was the most enriched pathway among the 334 upregulated genes, followed by the GO terms “sister chromatid cohesion”, “cell division”, “reciprocal meiotic recombination” and “regulation of transcription, DNA-templated” (Fig. 1d). In terms of the 472 downregulated genes, the GO terms “single strand break repair”, “embryonic organ development”, “cellular response to hypoxia”, “cholesterol biosynthesis process”, and “apoptotic process” were shown as the enriched pathways in cisplatin-resistant cells (Supplementary Fig. 1b). Among the upregulated genes in cisplatin-resistant cells, we focused on *HMGN5* (high mobility group nucleosome-binding domain 5) from GO term “transcription, DNA-templated” (GO:0006351) and *TEX11* (testis expressed 11) from GO term “reciprocal meiotic recombination” (GO:0007131), because *HMGN5* and *TEX11* were the most upregulated genes in NEC8-R versus NEC8 and TGCT-PDC-R versus TGCT-PDC cells (fold changes: 9.71 and 39.9, respectively). Both *HMGN5* and *TEX11* mRNAs were significantly upregulated in TGCT-PDC-R versus TGCT cells and NEC8-R versus NEC8 cells (Fig. 1e,f). Because cisplatin is a prototypic chemotherapy reagent that generates ICLs^8^, we showed that genes involved in the FA/BRCA pathway (e.g., *BRCA1*, *FANCD2*, *RAD51C*, and *RAD51*) were significantly upregulated in cisplatin-resistant cells as analyzed by qRT-PCR (Supplementary Fig. 1c).

### Silencing of HMGN5 and TEX11 inhibits cisplatin resistance in cisplatin-refractory TGCT cells

**Figure 2:**
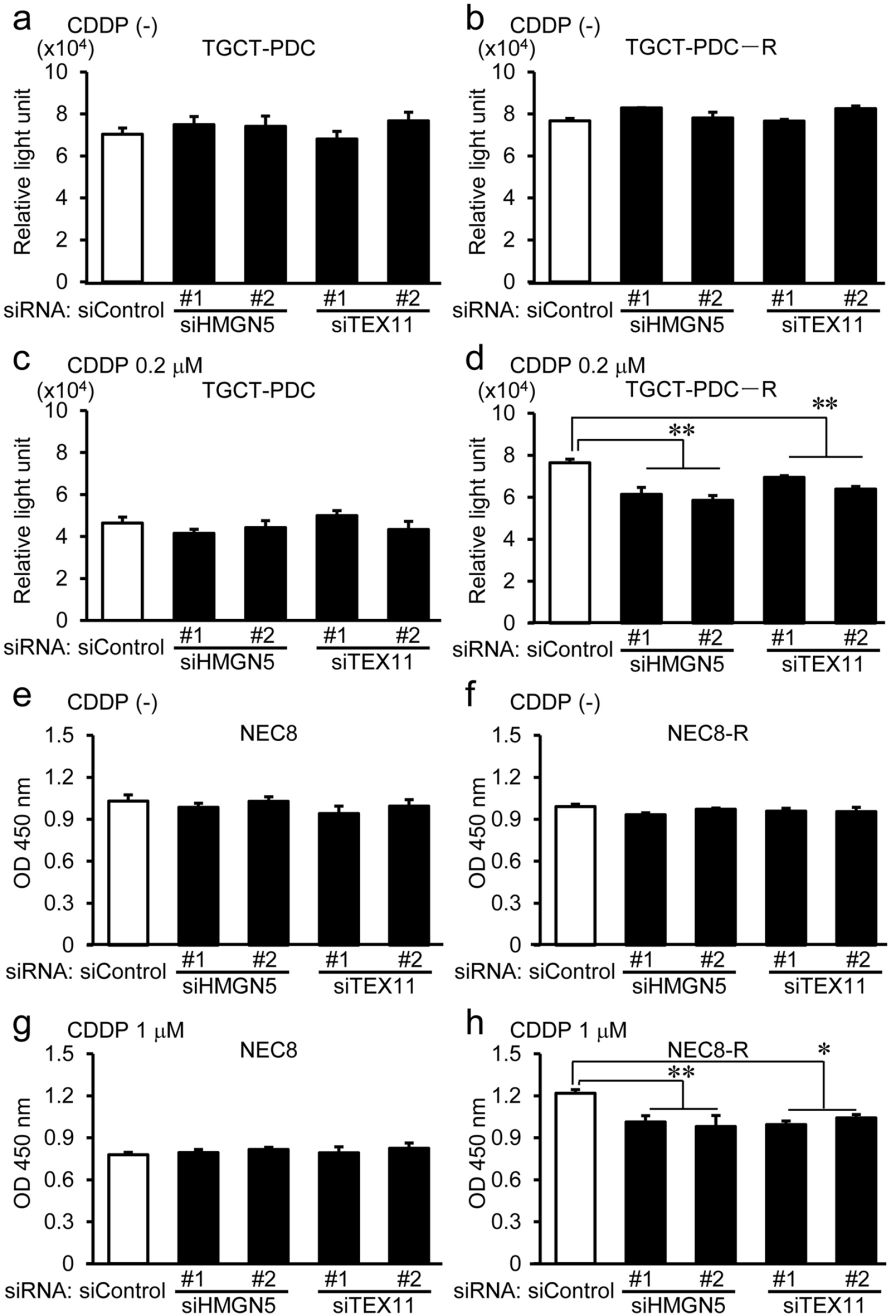
Silencing of HMGN5 and TEX11 attenuates the proliferation of cisplatin-resistant TGCT-PDC and NEC8 cells in the presence of cisplatin. **(a, b)** Effects of control siRNA (siControl), *HMGN5*-specific siRNAs (siHMGN5 #1 and #2), or *TEX11*-specific siRNAs (siTEX11 #1 and #2) on the viability of parental TGCT-PDC **(a)** and cisplatin-resistant TGCT-PDC-R **(b)** cells analyzed by quantitation of intracellular ATP content in the absence of cisplatin. Results are shown as mean ± SE (n = 4). **(c, d)** *HMGN5*- and *TEX11*-specific siRNAs repress the viability of TGCT-PDC-R cells **(d)** but not of TGCT-PDC cells **(c)** treated with cisplatin (0.2 μM). **(e, f)** Effects of indicated siRNAs on the viability of parental NEC8 **(e)** and cisplatin-resistant NEC8-R **(f)** cells analyzed by WST-8 cell proliferation assay in the absence of cisplatin. **(g, h)** *HMGN5*- and *TEX11*-specific siRNAs repress the viability of NEC8-R cells **(g)** but not of NEC8 cells **(h)** treated with cisplatin (0.2 μM). *, *P* < 0.05; **, *P* < 0.01.

We next assessed whether HMGN5 and TEX11 contribute to TGCT cell proliferation. Transfection of *HMGN5*- or *TEX11*-specific siRNAs could significantly downregulate their corresponding target mRNA levels compared to that of the control siRNA (siControl) in TGCT-PDC and NEC8 cells (Supplementary Fig. 2a, c) as well as their cisplatin-resistant cells (Supplementary Fig. 2b, d). While these siRNAs targeting *HMGN5* and *TEX11* did not substantially modulate the proliferation of parental TGCT-PDC (Fig. 2a,c) and NEC8 (Fig. 2e,g) cells compared to siControl with or without cisplatin treatment, it is notable that the specific siRNAs significantly repressed the proliferation of cisplatin-resistant cells compared to siControl only in the presence of cisplatin (Fig. 2d,h), but not in the absence of cisplatin (Fig. 2b,f).

### TEX11 knockdown enhances cisplatin-induced apoptosis and DNA damage in TGCT cells

HMGN family proteins have been known to interact with chromatin DNA and optimize DNA processes^24^. In terms of HMGN5, the silencing has been shown to promote chemosensitivity of human bladder cancer cells to cisplatin^25^. In contrast, the role of the meiosis-related gene TEX11 in TGCT tumorigenesis or cisplatin resistance has not been defined. We thus investigated whether TEX11 modulates cisplatin-induced apoptosis or DNA damage response in TGCT cells.

**Figure 3:**
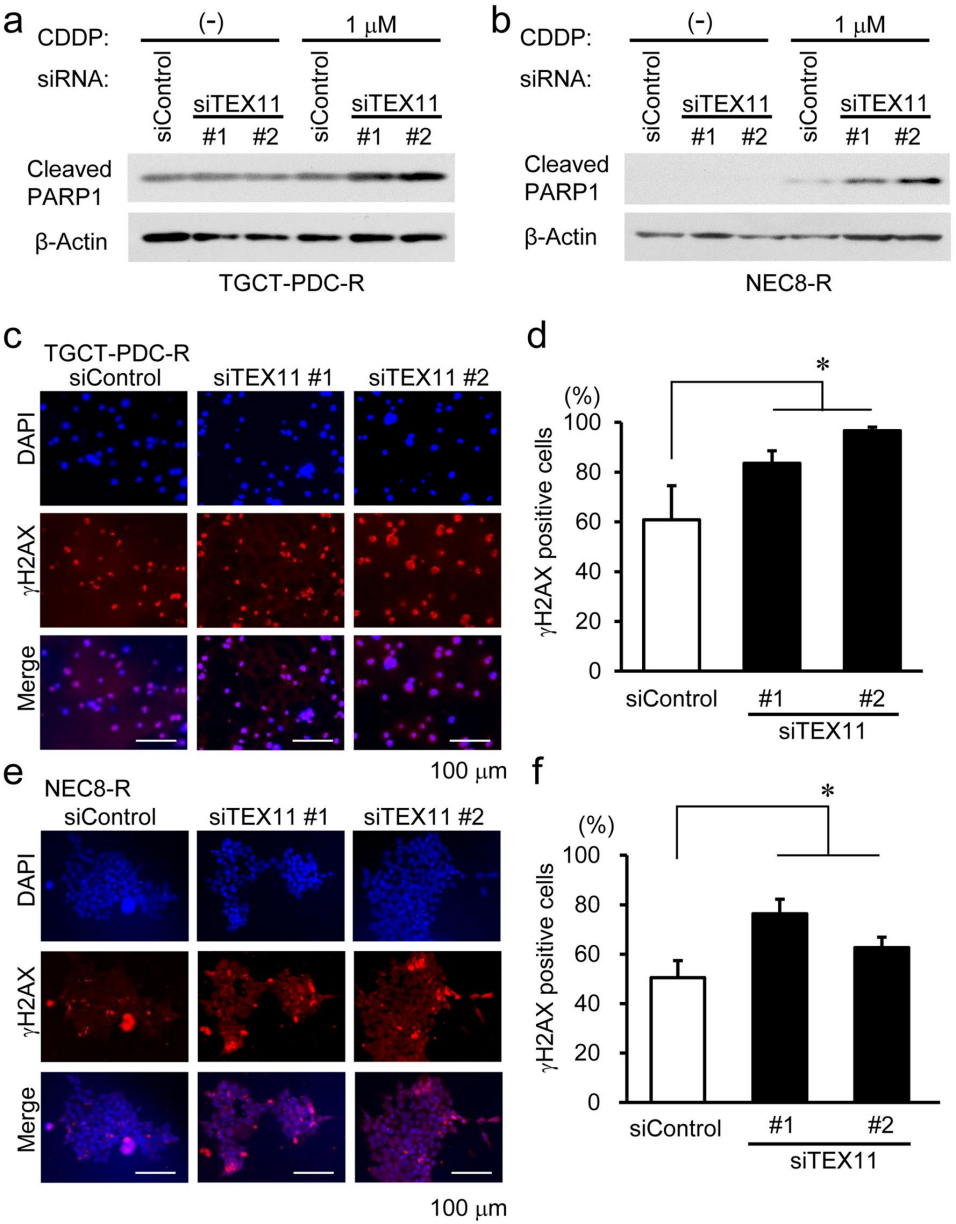
Effects of TEX11 expression on cisplatin-induced apoptosis and DNA double-strand break (DSB) marker γH2AX expression in TGCT cells. **(a, b)** Immunoblotting of cleaved PARP1 in cisplatin-resistant TGCT-PDC-R **(a)** and NEC8-R **(b)** cells transfected with control (siControl) or *TEX11*-specific siRNAs (siTEX11 #1 and #2), without or with cisplatin treatment 48 h after siRNA transfection. β-actin was used as a loading control. **(c)** Representative γH2AX immunohistochemical staining in TGCT-PDC-R cells transfected with indicated siRNAs, followed by cisplatin treatment 6 h after siRNA transfection. **(d)** Percentages of γH2AX-positive cells among the examined TGCT-PDC-R cells treated with the indicated siRNAs. The results are shown as mean percentage ± SE. **(e)** Representative γH2AX immunohistochemical staining in NEC8-R cells transfected with indicated siRNAs in the presence of cisplatin. **(f)** Percentages of γH2AX-positive cells among the examined NEC8-R cells treated with the indicated siRNAs. *, *P* < 0.05.

In both cisplatin-resistant TGCT-PDC-R and NEC8-R cells, immunoblot analysis showed that TEX11 silencing substantially increased the amounts of cleaved PARP1 protein in the presence of cisplatin (Fig. 3a,b). To further reveal whether TEX11 expression affects cisplatin‐induced DNA damage response, the phosphorylation of the serine 139 residue of the histone variant H2AX (γH2AX) was evaluated as a DSB marker in cisplatin-resistant TGCT cells treated with either *TEX11*-specific siRNAs or control siRNA in the presence of cisplatin. The immunofluorescence-based assay showed that the percentages of γH2AX positive cells significantly increased in both TGCT-PDC-R (Fig. 3c,d) and NEC8-R (Fig. 3e,f) cells treated with *TEX11*-specific siRNAs compared to those treated with siControl. Moreover, cell cycle profiling for TGCT-PDC-R and NEC8-R cells revealed that the *TEX11*-specific siRNAs significantly reduced the percentages of cell population in S phase in the presence but not in the absence of cisplatin treatment (Supplementary Fig. 3). These results indicate that TEX11 knockdown substantially enhances cisplatin-induced apoptosis and DNA damage even in cisplatin-resistant TGCT cells.

### TEX11 rescues cisplatin-induced DNA damage in TGCT cells

We next examined whether TEX11 overexpression elicits cisplatin resistance in cisplatin-sensitive TGCT cells. In NEC8 cells transfected with TEX11 expression plasmid, the levels of *TEX11* mRNA and TEX11 protein were substantially increased (Supplementary Fig. 4a, b). While the exogenous expression of TEX11 did not alter the viability of NEC8 cells compared to that of the control vector in the absence of cisplatin, TEX11 overexpression significantly recovered cell viability in the presence of cisplatin (Supplementary Fig. 4c). Notably, in the presence of cisplatin, the expression level of cleaved PARP1 protein was substantially decreased in cells with TEX11 overexpression compared to cells transfected with control vector (Supplementary Fig. 4d). Furthermore, the percentage of γH2AX-positive cells was significantly decreased in NEC8 cells overexpressing TEX11 (Supplementary Fig. 4e, f). Taken together, these results suggest that TEX11 may play a protective role in cisplatin-induced apoptosis and DSB repair.

### TEX11 knockdown of cisplatin-resistant TGCT cells reduces in vivo tumorigenicity

**Figure 4:**
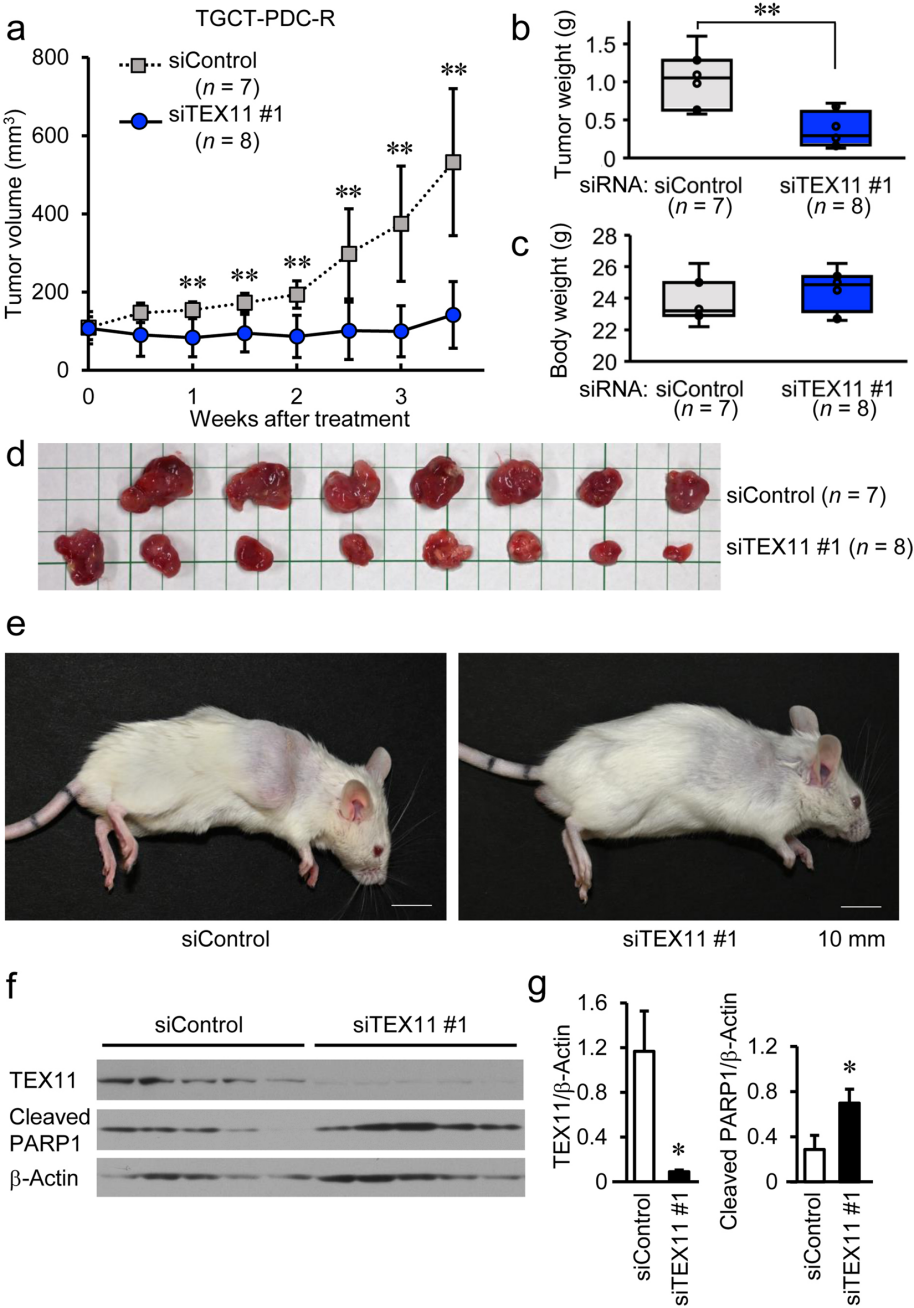
TEX11-specific siRNA injection significantly represses the development of TGCT-PDC-R-derived xenograft tumors in severely immunodeficient mice treated with cisplatin. **(a)** Volumes of tumors generated by TGCT-PDC-R cells in 7-week male NOD/SCID mice treated with intratumoral injection of control (siControl) or *TEX11*-specific (siTEX11) siRNAs plus intraperitoneal cisplatin administration. **(b)**Weights of tumors generated by TGCT-PDC-R in mice treated with injection of indicated siRNAs plus cisplatin administration. **(c)** Body weights of tumor-bearing mice treated with injection of indicated siRNAs plus cisplatin administration. Results are shown as mean ± SE. **, *P* < 0.01. **(d)** Images of dissected tumors at the endpoint. **(e)** Representative images of tumor-bearing mice at the endpoint. **(f)** Immunoblotting of TEX11 and cleaved PARP1 in dissected TGCT-PDC-R tumors. β-actin was used as a loading control. **(g)** Relative expression of TEX11 and cleaved PARP1 normalized to β-actin expression analyzed by densitometry. Results are shown as mean ± SE (n = 5). *, *P* < 0.05, **, *P* < 0.01.

We further evaluated whether *TEX11*-specific siRNA can reduce in vivo tumor growth of cisplatin-resistant TGCT cells using a TGCT-PDC-R-derived xenograft model of NOD/SCID mice treated with cisplatin. When the average volume of xenografted tumors reached 100 mm^3^, the injection of either siControl or siTEX11 #1 was started twice weekly into the subcutaneous tumors along with cisplatin treatment. In both groups, 2 mg/kg cisplatin was intraperitoneally administered once a week. It is notable that siTEX11 #1 injection remarkably suppressed the growth of TGCT-PDC-R-derived tumors compared with siControl injection (Fig. 4a,b,d,e), while the body weights of mice were not substantially different among the 2 groups (Fig. 4c). In the dissected tumors treated with siTEX11, the expression levels of cleaved PARP1 protein were increased compared with those of siControl-treated tumors (Fig. 4f). It is also notable that PCNA protein levels were relatively decreased in the siTEX11-treated tumors compared with siControl-treated tumors (Supplementary Fig. 5), suggesting that TEX11 silencing may repress the tumor proliferative activity. Overall, the in vivo experiments suggested that *TEX11*-specific siRNA can be used as a potential therapeutic device for cisplatin-resistant TGCT.

## Discussion

In the present study, we established cisplatin-resistant TGCT cell cultures from patient-derived TGCT cells and the NEC8 EC cell line, and further generated a xenograft model derived from cisplatin-resistant patient-derived TGCT cells. Based on transcriptomic analysis, we identified protumorigenic *HMGN5* and meiosis-related *TEX11* as genes commonly upregulated in cisplatin-resistant TGCT cell cultures compared with their corresponding parental cells. Focusing on the contribution of TEX11 to cisplatin resistance in TGCT cells, we showed that TEX11 silencing significantly repressed the growth of cisplatin-resistant TGCT cells, whereas it significantly increased the amount of cleaved PARP1 and the percentages of γH2AX-positive cells under the cisplatin-treated conditions. Notably, *TEX11*-specific siRNA significantly reduced the in vivo growth of xenograft tumors derived from cisplatin-resistant TGCT-PDC-R cells under the condition of cisplatin administration. Our findings indicate that TEX11 plays a critical role in cisplatin resistance in TGCT.

HMGN5 is a nuclear protein that belongs to the HMGN protein family, a subfamily of HMG proteins^26^. HGMN5 is ubiquitously expressed in human tissues and its high expression has been reported in several malignancies^27^. HMGN5 has a long and highly acidic C-terminal region that preferentially interacts with histone H1^28^, and unfolds the chromatin compaction mediated by linker histones including histone H1^26^. HMGN5 may play a role in the cisplatin-induced transcriptional alterations, as histone H1 is highly reactive to cisplatin and generates histone H1-cisplatin–DNA ternary complex. In osteosarcoma cells, cisplatin induced HMGN5 expression and its overexpression reduced in vitro drug sensitivity^29^. Besides on-target cisplatin resistance, HMGN5 may also be involved in post-target resistance, as its knockdown in prostate cancer cells elicited apoptosis and G2-M cell cycle arrest, leading to the sensitization of ionizing radiation^30^. HIF1α can be a candidate transcription factor that increases *HMGN5* expression in TGCT as reported in metastatic osteosarcoma^31^. Consistently, HIF1α is a potential molecular target for conquering cisplatin resistance in ovarian cancers^32^ and can be a therapeutic target in TGCT based on our findings using patient-derived models^21^. We thus consider that HMGN5 overexpression may be a potential biomarker for cisplatin resistance in TGCT.

In terms of DNA DSB repair provoked by ICLs, a previous study using cisplatin-resistant EC cells revealed that HR pathways were enhanced, whereas the non-homologous end-joining (NHEJ) pathways were dampened^33^. In that study, HR factor FANCD2 expression was upregulated whereas NHEJ factor TP53-binding protein 1 (TP53BP1) expression was repressed in cisplatin-resistant EC cells. This tendency is also observed in our transcriptomic data, in which *FANCD2* was upregulated as validated by qRT-PCR (Supplementary Fig. 1c), while *TP53BP1* was downregulated by 0.91 and 0.53 fold changes in TGCT-PDC-R and NEC8-R cells, respectively, compared with their corresponding parental cells. Other HR protein genes such as *RAD51C* and *BRCA1* were also upregulated in both TGCT-PDC-R and NEC8-R cells (Supplementary Fig. 1c). RAD51C is a member of the RAD51 family involved in HR pathways, and its biallelic missense mutations with impaired formation of RAD51 foci in response to DNA damage can be pathogenic reasons for FA-like phenotype^34^. The loss-of-function of RAD51C due to pathogenic mutations can elicit the increased sensitivity to DNA ICL reagents^34^. Germline mutations impairing RAD51C functions are susceptibility factors for breast and ovarian cancers like BRCA1/2^35^. Overexpression of RAD51C in non-small cell lung cancer cells increased cisplatin resistance and radiotherapy, and high expression of RAD51C in non-small cell lung cancer tissues was significantly associated with poor prognoses of the patients^36^. We thus consider that the enhancement of HR pathways can also substantially contribute to cisplatin resistance in our TGCT models.

Besides the above-mentioned mitotic HR proteins, it is notable that meiotic synapsis-related TEX11 was remarkedly upregulated in cisplatin-resistant TGCT cells. In DSB repair responses, the complex of meiotic recombination 11 homolog 1 (MRE11), ATP-binding cassette–ATPase (RAD50), and phosphopeptide-binding Nijmegen breakage syndrome protein 1 (NBS1/NBN) plays a central role in the formation of multifunctional DNA damage response machinery^37^. The MRE11-RAD50-NBS1 complex activities are also involved in various steps of programmed meiotic DSBs, including the initiation, SPO11 processing, and DSB repair^38^. TEX11/ZIP4H was initially identified as an NBS1-interacting protein with a significant structural similarity to the *S. cerevisiae* and *A. thaliana* Zip4 orthologs, based on a yeast two-hybrid screen of a human testis cDNA library^39^. In mice, TEX11/ZIP4H deficiency resulted in the perturbation of meiotic DSB repair and crossing over^39^. In infertile men, hemizygous *TEX11* mutations on chromosome Xq13.2 have been identified as a cause of meiotic arrest and azoospermia^40^. While the involvement of meiosis-related protein TEX11 in the mitotic processes remains elusive, the downregulation of *TEX11* was reported as a feature of gene expression profiles in early onset colorectal cancer patients compared with healthy controls^41^. Considering that genetic instability is a critical factor for the initiation of colorectal cancers, the reduced expression of TEX11 may lead to the impairment of DSB repair machinery. Intriguingly, our findings may indicate that the overexpression of TEX11 in TGCT cells results in the enhancement of DSB repairs exogenously induced by cisplatin as well as in the repression of apoptosis, suggesting that TEX11 might be involved in both on-target and post-target resistance. Furthermore, aberrant gene expression of cancer/testes (CT) antigens is a feature often observed in cancers^42,43^. For example, meiosis-related CT antigen HORMAD1 promotes DSB resection in lung cancer cells treated with cisplatin and overexpression of *HORMAD1* has been shown in clinical lung cancer tissues based on The Cancer Genome Atlas (TCGA) database^44^. Our findings together with previous reports may indicate that some meiotic HR-related factors can play a pathogenic role in chemoresistance in cancers by modulating DNA repair responses including mitotic HR pathways.

In terms of the transcriptional regulation of *TEX11*, we assume that SOX30 can be a candidate transcription factor that upregulates TEX11 in cisplatin-resistant TGCT cells. In the present study, *SOX30* was upregulated by 2.12 and 2.38 fold changes in TGCT-PDC-R versus TGCT-PDC and NEC8-R versus NEC8, respectively. Sox30 is a critical transcription factor in mouse spermatogenesis^45^, and *Tex11* expression in *Sox30* knockout mouse testes at step 3 round spermatid stage was basically null whereas that in wild-type mouse testes was substantial (> 3.5 fragments per kilobase of exon per million reads mapped) based on GSE113073 RNA-seq data retrieved from Gene Expression Omnibus^46^. Intriguingly, the TGCT RNA-sequencing dataset of TCGA PanCancer Atlas revealed that the coexpression correlation between *TEX11* and *SOX30* may be substantial as the Spearman’s correlation is 0.508 (*p*-value: 8.24e-11)[ https://www.cbioportal.org/].

Our transcriptomic data also showed the enriched pathways among the downregulated genes in cisplatin-resistant TGCT cells. Although the contribution of some reduced pathways to cisplatin resistance remains to be studied such as single strand break repair and embryonic organ development, it is notable that some genes involved in the apoptotic process were downregulated in cisplatin-resistant cells. Several pathways have been shown to be involved in the reduction of apoptosis in cisplatin-resistant TGCT, such as the elevated expression of p53 antagonist MDM2 and the activation of PI3K/Akt pathway^47^. Intriguingly, metabolic reprogramming has been also reported in cisplatin-resistant ovarian cancer cells, including the elevated activity of oxidative phosphorylation and the reduction of cholesterol biosynthesis^48,49^. Because genes involved in hypoxia response were also downregulated in the cisplatin-resistant TGCT cells, it remains to be elucidated whether the drug-induced metabolism can be another therapeutic target for the disease.

Patient-derived TGCT models such as patient-derived xenograft (PDX)^50–52^ have been recently used to evaluate chemosensitivity. In cisplatin-resistant TGCT PDX models, combined treatment with mTORC1/2 inhibitor and cisplatin led to the reduction of tumor growth^53^, validating that PI3K/Akt/mTORC pathway is one of the reasons for cisplatin resistance in TGCT. PDX models are preclinically useful for the prediction of tumor response to drugs because they recapitulate the pathophysiological features of patients’ tumors, although the maintenance of PDX models needs more resources than that of cell cultures because of the requirement of the in vivo passages^54^. The patient-derived spheroid cultures of TGCT cells that we used in the present study initially exhibit a characteristic of cancer stem-like cells (CSCs) because the model abundantly expressed CSC markers such as NANOG and SOX2, and the TGCT cultures can be easily applied to in vivo models^21^. While TEX11 and HMGN5 were commonly upregulated in cisplatin-resistant patient-derived TGCT-PDC-R and cell line-derived NEC8-R cells, it remains to be elucidated whether the difference of gene expression profiles in these cells is individually associated with chemoresistance and tumor aggressiveness.

Clinically, it is interesting to elucidate whether high expression of TEX11 and HMGN5 can be predictive refractory biomarkers for cisplatin in TGCTs prior to chemotherapy. Based on the RNA-sequencing dataset of TGCT cohort with 144 patients in TCGA database, 4 and 6% of the patient samples are categorized as mRNA high for *TEX11* and *HMGN5*, respectively. Among the cohort, TGCT samples with both high levels of *TEX11* and *HMGN5* mRNA are recurrent in progressed tumors. While no substantial correlation of coexpression between *TEX11* and *HMGN5* was observed in the TCGA dataset, future investigations with a larger number of patients will reveal the clinical relevance of these genes in the management of the advanced disease.

## Methods

### Cell culture

TGCT patient-derived cell (TGCT-PDC) spheroid culture was established as previously described^21^, from primary EC tumor with CD30 expression, resected from a patient aged 26 years at operation with informed consent based on the protocol #1363-IX approved by Saitama Medical Center Institutional Review Board. Human EC cell line NEC8 was purchased from Riken BioResource Center and cultured in RPMI 1640 (Nacalai Tesque) with 10% FBS, penicillin (100 units/mL), and streptomycin (100 μg/mL) at 37 °C in a humidified atmosphere of 5% CO_2_. Cell authentication was confirmed by short tandem repeat (STR) profiling.

### Generation of cisplatin-resistant cells

Cisplatin-resistant TGCT-PDC and NEC8 cells (TGCT-PDC-R and NEC8-R cells, respectively) were generated from the corresponding parental cells by stepwise increase in concentrations of cisplatin (Wako) for > 4 months, based on the protocol by Tada et al*.*^55^. The final maintenance concentrations of cisplatin were 0.4 µM and 1.0 µM for TGCT-PDC-R and NEC8-R cells, respectively.

### Microarray and pathway analysis

Expression microarray analysis was performed using the platform of GeneChip Human Gene 1.0 ST Array (Affymetrix) according to the manufacturer’s protocol. Data were analyzed using Affymetrix Microarray Suite software. Pathway analysis was carried out based on the web-accessible program the Database for Annotation, Visualization and Integrated Discovery (DAVID) Bioinformatics Resources v6.8 (https://david.ncifcrf.gov/).

### SiRNAs

siRNAs against *HMGN5* and *TEX11* were designed using Enhanced siDirect siRNA design algorithm provided by RNAi Inc. and synthesized by Japan Bio Services Co., LTD. Negative control siRNA (siControl) with no homology to known gene targets in mammalian cells was from RNAi Inc. Sequences of siRNAs are listed in Supplemantary Table 3.

### Quantitative reverse transcription polymerase chain reaction

Total RNAs were extracted from cells using Sepasol-RNA I Super G (Nacalai Tesque) and first stranded cDNAs were synthesized using PrimeScript II Reverse Transcriptase (Takara) with oligo dT primer. Quantitative RT-PCR (qRT-PCR) was carried out using KAPA SYBR FAST (KAPA Biosystems) and sets of gene-specific primers on StepOnePlus Real-Time PCR System (Thermo Fisher Scientific). Relative RNA levels were analyzed by the ΔΔCt method according to the manufacturer’s protocol and normalized to the values of *ACTB*. Student’s *t*-test was used for statistical analysis and *P* < 0.05 was considered statistically significant. Sequences of primers used in this study are listed in Supplementary Table 4.

### Cell viability assay

TGCT-PDC and TGCT-PDC-R cells were seeded at 10,000 cells/well in 96‐well plates and simultaneously transfected with indicated siRNAs at a final concentration of 30 nM using Lipofectamine RNAiMAX reagent (Invitrogen). Six hours after transfection, medium was added to increase the concentration of cisplatin to 0.2 µM. Cell viability was measured by quantitation of intracellular ATP content using CellTiter-Glo 3D Cell Viability Assay Kit (Promega) at 96 h after transfection. After 30 min of cell lysis, chemiluminescent values were measured using the TriStar2 S LB 942 Multimode Reader (Berthold Technologies). NEC8 and NEC8-R cells were seeded at 2,000 cells/well in 96‐well plates and 24 h later transfected with indicated siRNAs at a final concentration of 10 nM using Lipofectamine RNAiMAX reagent. Twenty-four hours after transfection, medium was added to increase the concentration of cisplatin to 1.0 µM. At 72 h after transfection, 10 μL of a Cell Count Reagent SF, a reagent containing 2-(2-methoxy-4-nitrophenyl)-3-(4-nitrophenyl)-5-(2,4-disulfophenyl)-2H-tetrazolium, monosodium salt (WST-8) (Nacalai Tesque) was added to each well and the cells were incubated for 4 h at 37 °C. Absorbance of the plates was read on Multiskan FC Photometer (Thermo scientific) at a wavelength of 450 nm.

### Cell cycle analysis

TGCT-PDC-R and NEC8-R cells were transfected with indicated siRNAs at a final concentration of 30 and 10 nM, respectively, using Lipofectamine RNAiMAX reagent (Invitrogen), and cisplatin was added to the medium 24 h after transfection at a final concentration of 1 µM. Cells were fixed in 70% ethanol 48 h after siRNA transfection, followed by propidium iodide staining (10 μg/mL) as previously described^56^. Cell cycle distribution for each sample was analyzed by fluorescence-activated cell sorting (FACS) (BD Accuri C6 Flow Cytometer; BD Sciences) based on DNA content, and the percentages of cells in the G1, S, and G2/M phases of the cell cycle were evaluated by BD Accuri C6 software.

### Transient overexpression of TEX11

The full-length cDNA of *TEX11* was amplified from the total RNA of NEC8 cells using PCR and ligated into the pcDNA3 vector. The TEX11 expression plasmid was transduced into NEC8 cells using Lipofectamine 3000 Transfection kit (Invitrogen).

### Antibodies

Rabbit polyclonal antibody to TEX11 (NBP2-94,329) was purchased from Novus Biologicals. Rabbit monoclonal antibodies to cleaved PARP1 (clone: E51) and phospho-histone H2A.X (Ser139) (clone: 20E3) were purchased from Abcam and Cell signaling technology, respectively. Mouse monoclonal antibody to β-actin (clone: AC-74) was purchased from Sigma-Aldrich.

### Immunoblotting

Cells were lysed in RIPA buffer (50 mM Tris–HCl, pH 8.0, 150 mM NaCl, 1% Triton-X 100, supplemented with 1 mM phenylmethylsulfonyl fluoride before usage). Extracted proteins were separated with SDS‐PAGE and blotted on PVDF membrane, followed by antibody reactions^56^.

### Immunofluorescence staining

TGCT-PDC-R spheroids transfected with indicated siRNAs were dissociated with Accutase (Nacalai Tesque) into single-cell suspensions and attached to the glass slide using Smear Gel (GenoStaff) according to the manufacturer’s instruction. Cells in Smear Gel were fixed in 4% paraformaldehyde (PFA) 6 h after addition of cisplatin, permeabilized with 1% Triton-X (Nacalai Tesque) for 10 min, rinsed with Tris-buffered saline (TBS), blocked with 10% FBS. After blocking, the slides were incubated with the anti-phospho-histone H2A.X antibody (1:400) for overnight at 4 °C. Then the slides were incubated in secondary Cy3-labeled anti-rabbit IgG antibodies (1:400, Jackson ImmunoResearch Laboratories). Nuclei were counterstained with DAPI and visualized under fluorescent microscope (All-in-One Fluorescence Microscope, KEYENCE). Cells per field were counted using imaging software Bz-X Analyzer 1.4.1.1 (KEYENCE).

### In vivo tumor formation and siRNA treatment

All animal experiments were approved by the Animal Care and Use Committee of Saitama Medical University and conducted following the institutional Guidelines and Regulations. Male NOD/SCID mice (C.B-17/Icr-scid/scidJcl) were purchased from CREA Japan Inc (Tokyo, Japan). TGCT-PDC-R cells (100,000 cells per mouse) were mixed with an equal volume of Matrigel (Corning) and injected subcutaneously into the right flank of 7-week-old male NOD/SCID mice. TGCT-PDC-R cells were inoculated into 20 male mice, and the development of xenograft tumors were obesrved in 15 mice. Then, we randomly assigned these 15 mice to the siControl (n = 7) or siTEX11 #1 groups (n = 8). When the average tumor volume exceeded 100 mm^3^, siControl or siTEX11 #1 (5 μg each) prepared with 4 µL GeneSilencer reagent (Genlantis) were directly injected into the tumors twice a week as described previously^56^. Furthermore, all mice were intraperitoneally administrated with 2 mg/kg cisplatin dissolved in 0.15 mL natural saline once a week. Three dimensions of tumor were measured with calipers twice a week, and tumor volumes were calculated using the following formula: 0.52 × largest dimension × intermediate dimension × shortest dimension. At the endpoint of the experiment, the tumors were dissected from the mice, homogenized in RIPA buffer, and subjected to immunoblot analysis with anti-TEX11, anti-cleaved PARP1, and anti-β-actin antibodies.

### Statistical analysis

Statistical analysis was performed using Microsoft Excel for Mac 16.54 (Microsoft Corporation) or JMP 15.0.0 (SAS Institute) with a Student’s *t*-test for pairwise comparison and two-way ANOVA for multiple comparisons.

### Approval for animal experiments

All animal experiments were approved by the Animal Care and Use Committee of Saitama Medical University (protocol # 3124) and conducted in accordance with the institutional guidelines and regulations. All methods are reported in accordance with ARRIVE guidelines for the reporting of animal experiments.

### Approval for human experiments

This study abided by the Declaration of Helsinki principles and all methods on humans were carried out in accordance with Saitama Medical Center Institutional Review Board experimental guidelines and regulations for human subjects (protocol #1363-IX). The informed consent was obtained from the patient with TGCT, whose tumor tissue was used for the establishment of TGCT-PDC cells.

## Supplementary Information

Supplementary Information 1.

Supplementary Information 2.

## Data Availability

Microarray data generated during the current study are available in the Gene Expression Omnibus (GEO) database with the accession number GSE197347.

## References

[1] Cheng L (2018). Testicular cancer. Nat. Rev. Dis. Primers.

[2] McHugh DJ, Feldman DR (2018). Conventional-dose versus high-dose chemotherapy for relapsed germ cell tumors. Adv. Urol..

[3] Galluzzi L (2012). Molecular mechanisms of cisplatin resistance. Oncogene.

[4] Eastman A (1986). Reevaluation of interaction of cis-dichloro (ethylenediamine)platinum(II) with DNA. Biochemistry.

[5] Huang JC, Zamble DB, Reardon JT, Lippard SJ, Sancar A (1994). HMG-domain proteins specifically inhibit the repair of the major DNA adduct of the anticancer drug cisplatin by human excision nuclease. Proc. Natl. Acad. Sci. USA.

[6] Clingen PH (2008). Histone H2AX phosphorylation as a molecular pharmacological marker for DNA interstrand crosslink cancer chemotherapy. Biochem. Pharmacol..

[7] Park HJ (2018). The PARP inhibitor olaparib potentiates the effect of the DNA damaging agent doxorubicin in osteosarcoma. J. Exp. Clin. Cancer Res..

[8] Niraj J, Färkkilä A, D'Andrea AD (2019). The Fanconi anemia pathway in cancer. Annu. Rev. Cancer Biol..

[9] Welsh C (2004). Reduced levels of XPA, ERCC1 and XPF DNA repair proteins in testis tumor cell lines. Int. J. Cancer.

[10] Usanova S (2010). Cisplatin sensitivity of testis tumour cells is due to deficiency in interstrand-crosslink repair and low ERCC1-XPF expression. Mol. Cancer.

[11] Hamer G, de Rooij DG (2018). Mutations causing specific arrests in the development of mouse primordial germ cells and gonocytes. Biol. Reprod..

[12] Hill RJ, Crossan GP (2019). DNA cross-link repair safeguards genomic stability during premeiotic germ cell development. Nat. Genet..

[13] Keeney S, Giroux CN, Kleckner N (1997). Meiosis-specific DNA double-strand breaks are catalyzed by Spo11, a member of a widely conserved protein family. Cell.

[14] Keeney S (1999). A mouse homolog of the Saccharomyces cerevisiae meiotic recombination DNA transesterase Spo11p. Genomics.

[15] Romanienko PJ, Camerini-Otero RD (2000). The mouse Spo11 gene is required for meiotic chromosome synapsis. Mol. Cell.

[16] Hanneman WH, Legare ME, Sweeney S, Schimenti JC (1997). Cisplatin increases meiotic crossing-over in mice. Proc. Natl. Acad. Sci. USA.

[17] Carofiglio F (2018). Repair of exogenous DNA double-strand breaks promotes chromosome synapsis in SPO11-mutant mouse meiocytes, and is altered in the absence of HORMAD1. DNA Repair (Amst).

[18] Berney DM (2016). Germ cell neoplasia in situ (GCNIS): evolution of the current nomenclature for testicular pre-invasive germ cell malignancy. Histopathology.

[19] Looijenga LH (2003). POU5F1 (OCT3/4) identifies cells with pluripotent potential in human germ cell tumors. Cancer Res..

[20] de Jong J (2008). Differential expression of SOX17 and SOX2 in germ cells and stem cells has biological and clinical implications. J. Pathol..

[21] Namekawa T (2020). HIF1α inhibitor 2-methoxyestradiol decreases NRN1 expression and represses in vivo and in vitro growth of patient-derived testicular germ cell tumor spheroids. Cancer Lett..

[22] Kapur P, Rakheja D (2021). Basic histopathologic assessment of germ cell tumors for clinic and research. Methods Mol. Biol..

[23] Cavallo F (2012). Reduced proficiency in homologous recombination underlies the high sensitivity of embryonal carcinoma testicular germ cell tumors to cisplatin and poly (adp-ribose) polymerase inhibition. PLoS ONE.

[24] Nanduri R, Furusawa T, Bustin M (2020). Biological functions of HMGN chromosomal proteins. Int. J. Mol. Sci..

[25] Gan Y (2017). Knockdown of HMGN5 increases the chemosensitivity of human urothelial bladder cancer cells to cisplatin by targeting PI3K/Akt signaling. Oncol. Lett..

[26] Rochman M, Malicet C, Bustin M (2010). HMGN5/NSBP1: a new member of the HMGN protein family that affects chromatin structure and function. Biochim. Biophys. Acta.

[27] Shi Z, Tang R, Wu D, Sun X (2016). Research advances in HMGN5 and cancer. Tumour Biol..

[28] Furusawa T, Cherukuri S (2010). Developmental function of HMGN proteins. Biochim Biophys Acta.

[29] Yang C, Gao R, Wang J, Yuan W, Wang C, Zhou X (2014). High-mobility group nucleosome-binding domain 5 increases drug resistance in osteosarcoma through upregulating autophagy. Tumour Biol..

[30] Su B (2015). HMGN5 knockdown sensitizes prostate cancer cells to ionizing radiation. Prostate.

[31] Xu E, Ji Z, Jiang H, Lin T, Ma J, Zhou X (2019). Hypoxia-inducible factor 1A upregulates HMGN5 by increasing the expression of GATA1 and plays a role in osteosarcoma metastasis. Biomed. Res. Int..

[32] Ai Z, Lu Y, Qiu S, Fan Z (2016). Overcoming cisplatin resistance of ovarian cancer cells by targeting HIF-1-regulated cancer metabolism. Cancer Lett..

[33] Caggiano C (2021). Testicular germ cell tumors acquire cisplatin resistance by rebalancing the usage of DNA repair pathways. Cancers (Basel).

[34] Vaz F (2010). Mutation of the RAD51C gene in a Fanconi anemia-like disorder. Nat. Genet..

[35] Meindl A (2010). Germline mutations in breast and ovarian cancer pedigrees establish RAD51C as a human cancer susceptibility gene. Nat Genet..

[36] Chen X (2016). High expression of Rad51c predicts poor prognostic outcome and induces cell resistance to cisplatin and radiation in non-small cell lung cancer. Tumour Biol..

[37] Syed A, Tainer JA (2018). The MRE11-RAD50-NBS1 complex conducts the orchestration of damage signaling and outcomes to stress in DNA replication and repair. Annu. Rev. Biochem..

[38] Richardson C, Horikoshi N, Pandita TK (2004). The role of the DNA double-strand break response network in meiosis. DNA Repair (Amst).

[39] Adelman CA, Petrini JH (2008). ZIP4H (TEX11) deficiency in the mouse impairs meiotic double strand break repair and the regulation of crossing over. PLoS Genet..

[40] Yatsenko AN (2015). X-linked TEX11 mutations, meiotic arrest, and azoospermia in infertile men. N. Engl. J. Med..

[41] Luo T, Wu S, Shen X, Li L (2013). Network cluster analysis of protein-protein interaction network identified biomarker for early onset colorectal cancer. Mol. Biol. Rep..

[42] Almeida LG (2009). CTdatabase: A knowledge-base of high-throughput and curated data on cancer-testis antigens. Nucleic Acids Res..

[43] Whitehurst AW (2014). Cause and consequence of cancer/testis antigen activation in cancer. Annu. Rev. Pharmacol. Toxicol..

[44] Gao Y (2018). The Cancer/Testes (CT) Antigen HORMAD1 promotes homologous recombinational DNA repair and radioresistance in lung adenocarcinoma cells. Sci. Rep..

[45] Feng CA, Spiller C, Merriner DJ, O'Bryan MK, Bowles J, Koopman P (2017). SOX30 is required for male fertility in mice. Sci Rep..

[46] Zhang D (2018). The transcription factor SOX30 is a key regulator of mouse spermiogenesis. Development.

[47] Jacobsen C, Honecker F (2015). Cisplatin resistance in germ cell tumours: models and mechanisms. Andrology.

[48] Matassa DS (2016). Oxidative metabolism drives inflammation-induced platinum resistance in human ovarian cancer. Cell Death Differ..

[49] Criscuolo D (2020). Cholesterol homeostasis modulates platinum sensitivity in human ovarian cancer. Cells.

[50] Juliachs M (2013). Effectivity of pazopanib treatment in orthotopic models of human testicular germ cell tumors. BMC Cancer.

[51] Piulats JM (2018). Orthoxenografts of testicular germ cell tumors demonstrate genomic changes associated with cisplatin resistance and identify PDMP as a resensitizing agent. Clin. Cancer Res..

[52] de Vries G (2020). Establishment and characterisation of testicular cancer patient-derived xenograft models for preclinical evaluation of novel therapeutic strategies. Sci. Rep..

[53] Rosas-Plaza X (2020). Dual mTORC1/2 inhibition sensitizes testicular cancer models to cisplatin treatment. Mol. Cancer Ther..

[54] Namekawa T, Ikeda K, Horie-Inoue K, Inoue S (2019). Application of prostate cancer models for preclinical study: advantages and limitations of cell lines, patient-derived xenografts, and three-dimensional culture of patient-derived cells. Cells.

[55] Tada Y (2011). Ectonucleoside triphosphate diphosphohydrolase 6 expression in testis and testicular cancer and its implication in cisplatin resistance. Oncol. Rep..

[56] Sato W (2018). Efp promotes in vitro and in vivo growth of endometrial cancer cells along with the activation of nuclear factor-κB signaling. PLoS ONE.

