# Supplementary material for: Testis-expressed gene 11 inhibits cisplatin-induced DNA damage and contributes to chemoresistance in testicular germ cell tumor
Source: Sci Rep. 2022 Nov 1;12:18423. doi: 10.1038/s41598-022-21856-3 (PMC9626550; doi:10.1038/s41598-022-21856-3)
Supplement: Supplementary file 1 — Supplementary Information 1. [file 41598_2022_21856_MOESM1_ESM.pdf]

Supplementary Information

Testis-expressed gene 11 inhibits cisplatin-induced DNA damage and contributes to chemoresistance in testicular germ cell tumor

Sachi Kitayama, Kazuhiro Ikeda, Wataru Sato, Hideki Takeshita, Satoru Kawakami, Satoshi Inoue & Kuniko Horie

Supplementary Figure 1: Identification of downregulated genes in cisplatin-resistant TGCT cells *versus* parental cells, and validation of HR-related gene expression in TGCT cells.
Supplementary Figure 2: Knockdown efficiency of siRNAs specific to *HMGN5* and *TEX11* in TGCT cells.
Supplementary Figure 3: Knockdown efficiency of TEX11-specific siRNAs on cell cycle profiling in cisplatin-resistant TGCT cells.
Supplementary Figure 4: TEX11 overexpression preserves TGCT cell viability in the presence of cisplatin and decreases cisplatin-induced apoptosis and γH2AX expression.
Supplementary Figure 5: TEX11 silencing represses the expression of proliferative marker PCNA in TGCT-PDC-R-derived xenograft tumors.
Supplementary Figure 6: Uncropped blot images in Figure 3a.
Supplementary Figure 7: Uncropped blot images in Figure 3b.
Supplementary Figure 8: Uncropped blot images in Figure 4f.
Supplementary Figure 9: Uncropped blot images in Supplementary Figure 4b.
Supplementary Figure 10: Uncropped blot images in Supplementary Figure 4d.
Supplementary Figure 11: Uncropped blot images in Supplementary Figure 5a.

Supplementary Tables are provided in a separated datasheet file.
Supplementary Table 1: List of 334 commonly upregulated genes in cisplatin-resistant TGCT-PDC-R and NEC8-R cells over their corresponding parental cells (fold change ≥1.5, fluorescence signal in cisplatin-resistant cells ≥5).
Supplementary Table 2: List of 472 commonly downregulated genes in cisplatin-resistant TGCT-PDC-R and NEC8-R cells over their corresponding parental cells (fold change ≤ 0.67 in cisplatin-resistant cells, fluorescence signal ≥ 5 in parental cells).
Supplementary Table 3: Sequences of siRNAs used in the present study.
Supplementary Table 4: Primers used in the present study.

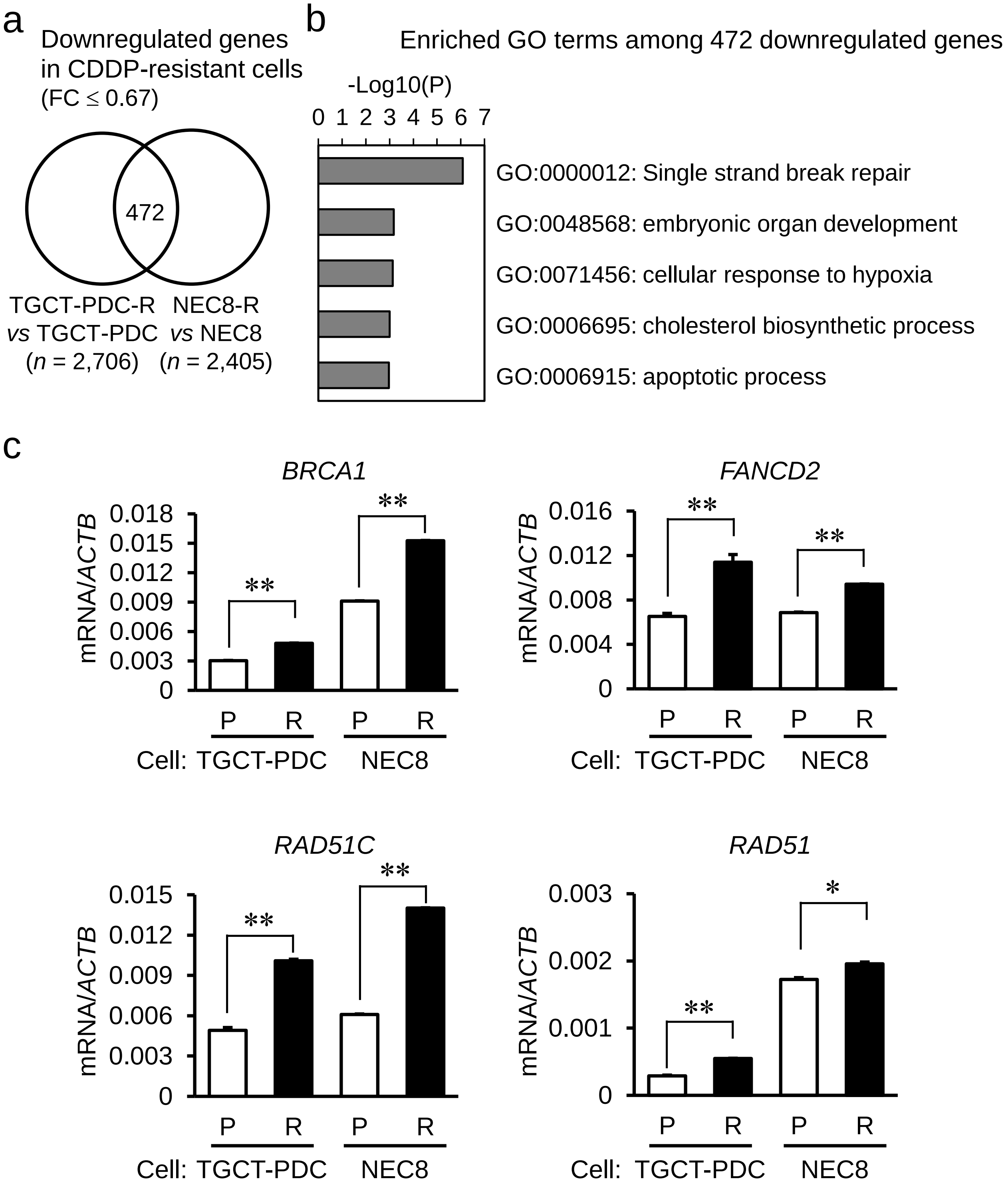

Supplementary Figure 1

Supplementary Figure 1: Identification of downregulated genes in cisplatin-resistant TGCT cells *versus* parental cells, and validation of HR-related gene expression in TGCT cells.

(a) Microarray analysis identified 472 overlapping genes commonly downregulated by ≤0.67 fold in cisplatin-resistant cells compared to the corresponding parental cells (with a fluorescence signal ≥5 in parental cells).

(b) Top 5 signaling pathways enriched among the 472 commonly downregulated genes in cisplatin-resistant TGCT-PDC and NEC8 cells based on Gene Ontology Term.

(c) Expression of HR-related genes *BRCA1*, *FANCD2*, *RAD51C*, and *RAD51* in TGCT cells validated by qRT-PCR. Indicated mRNA levels were normalized to *ACTB* mRNA levels. P, parental cells. R, cisplatin-resistant cells. Results are shown as mean ± SE (n = 3). *, $P < 0.05$; **, $P < 0.01$.

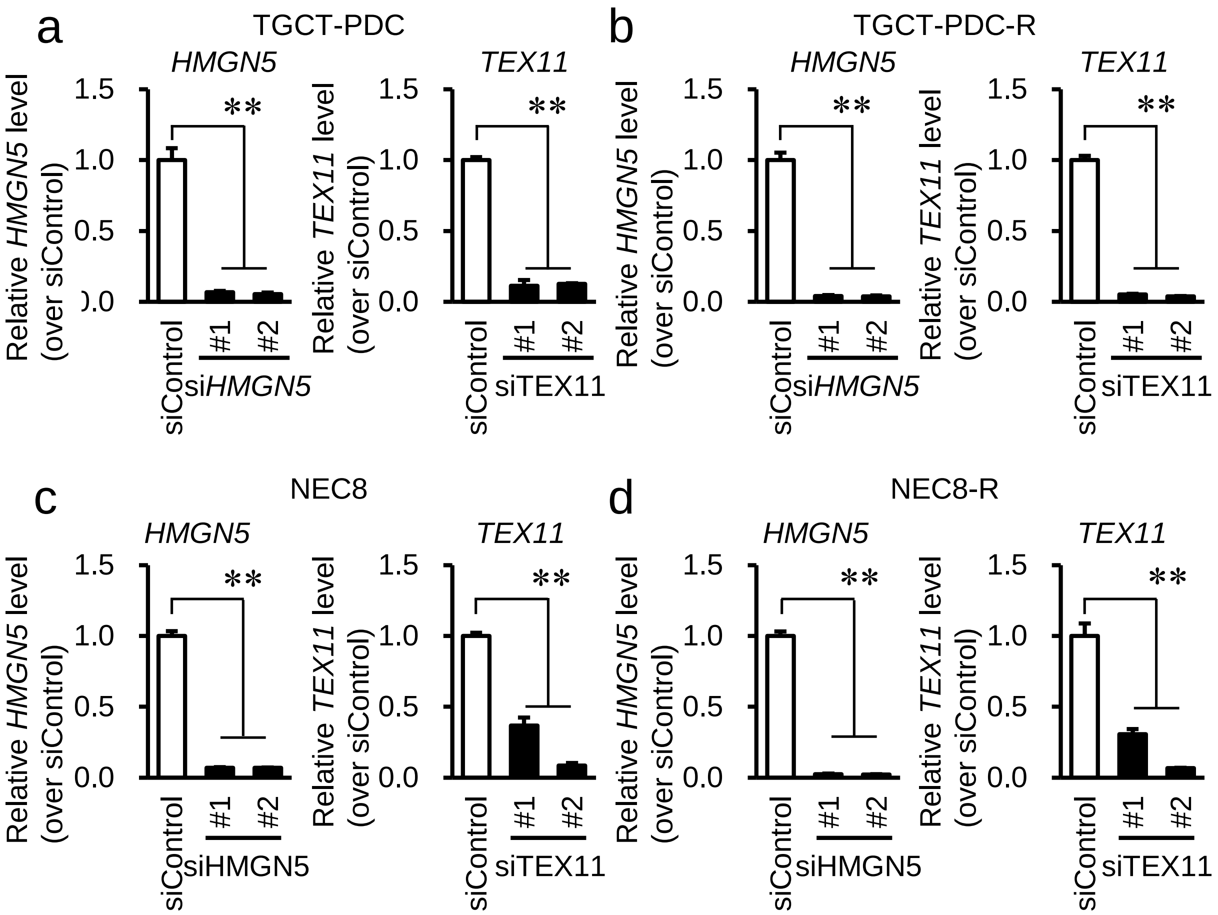

Supplementary Figure 2

Supplementary Figure 2: Knockdown efficiency of siRNAs specific to *HMGN5* and *TEX11* in TGCT cells.

siRNAs specific to *HMGN5* (siHMGN5 #1 and #2) and *TEX11* (siTEX11 #1 and #2) significantly downregulate *HMGN5* and *TEX11* mRNA levels, respectively, in parental TGCT-PDC **(a)** and NEC8 **(c)** cells as well as in cisplatin-resistant TGCT-PDC-R **(b)** and NEC8-R **(d)** cells. Indicated mRNA levels were normalized to *ACTB* mRNA levels. Results are shown as mean ± SE. *, $P < 0.05$; **, $P < 0.01$.

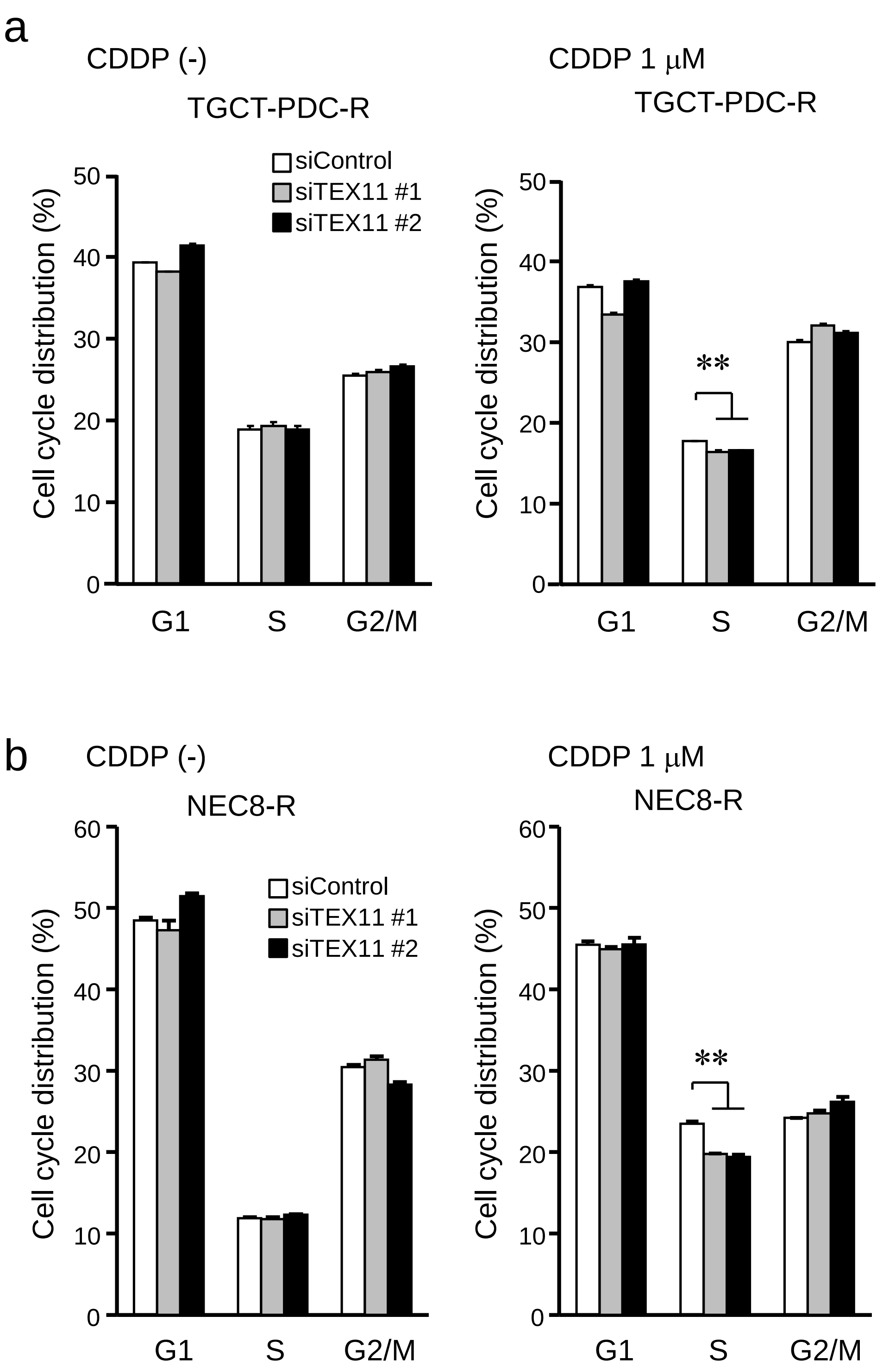

Supplementary Figure 3

Supplementary Figure 3: Knockdown efficiency of TEX11-specific siRNAs on cell cycle profiling in cisplatin-resistant TGCT cells.

(a, b) Effects of control siRNA (siControl) or *TEX11*-specific siRNAs (siTEX11 #1 and #2) on the cell cycle profiling of cisplatin-resistant TGCT-PDC-R (**a**) and NEC8-R (**b**) cells in the absence (left panels) and the presence of cisplatin (right panels) analyzed by FACS. Results are shown as mean ± SE (n = 4). **, P < 0.01.

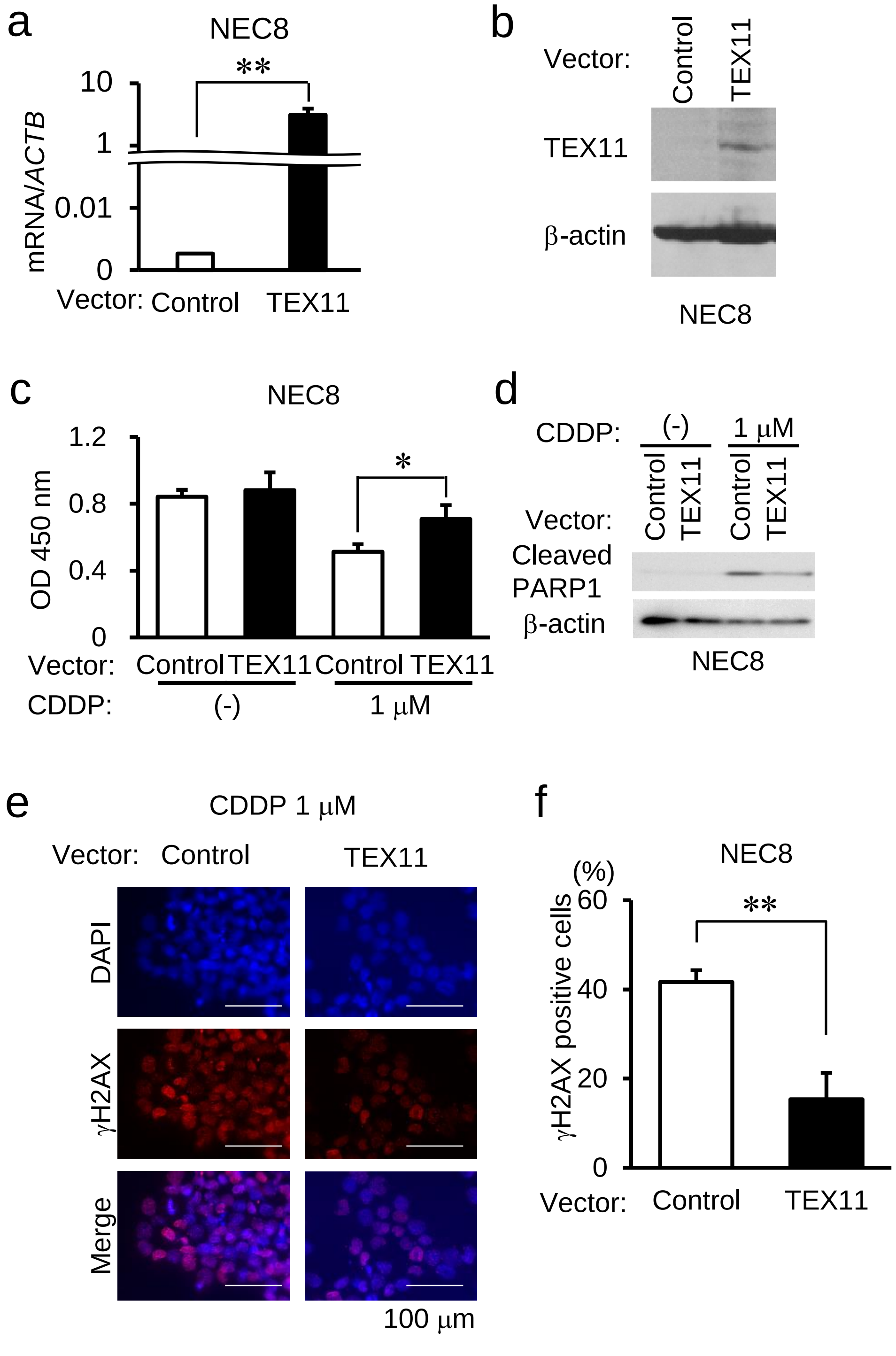

Supplementary Figure 4

Supplementary Figure 4: TEX11 overexpression preserves TGCT cell viability in the presence of cisplatin and decreases cisplatin-induced apoptosis and γH2AX expression.

(a) *TEX11* mRNA levels in NEC8 cells transfected with control or *TEX11* expression vectors analyzed by qRT-PCR.

(b) Immunoblotting of TEX11 in NEC8 cells transfected with control or *TEX11* expression vectors. β-actin was used as a loading control.

(c) NEC8 cell viability transfected with control or *TEX11* expression vectors analyzed by WST-8 cell proliferation assay without or with cisplatin treatment.

(d) Immunoblotting of cleaved PARP1 in NEC8 cells transfected with control or *TEX11* expression vectors without or with subsequent cisplatin treatment 48 hours after vector transfection. β-actin was used as a loading control.

(e) Representative γH2AX immunostaining in NEC8 cells transfected with control or *TEX11* expression vectors in the presence of cisplatin. Cells were treated with cisplatin (1 μM) for 6 hours after vector transfection.

(f) Percentages of γH2AX-positive cells among the examined NEC8 cells transfected with control or *TEX11* expression vectors treated with cisplatin (1 μM). Results are shown as mean percentage ± SE. *, $P < 0.05$, **, $P < 0.01$.

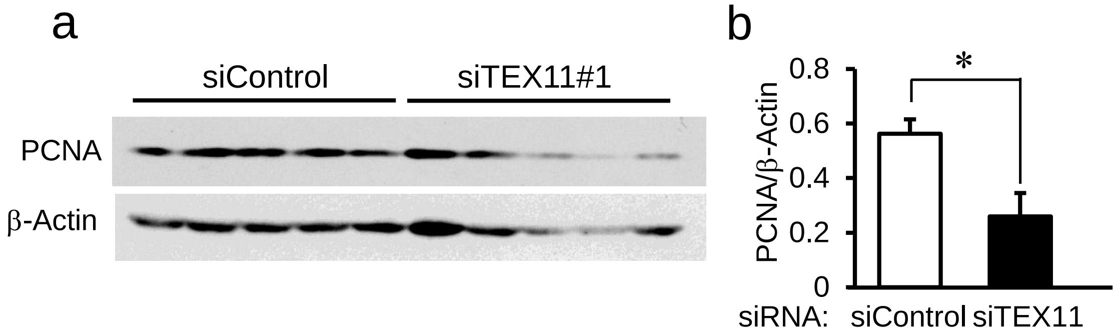

Supplementary Figure 5

Supplementary Figure 5: TEX11 silencing represses the expression of proliferative marker PCNA in TGCT-PDC-R-derived xenograft tumors.

(a) Immunoblotting of PCNA in dissected TGCT-PDC-R tumors treated with TEX11-specific or control siRNAs.

(b) Relative expression of PCNA normalized to β-actin expression analyzed by densitometry. Results are shown as mean ± SE (n = 5). *, $P < 0.05$.

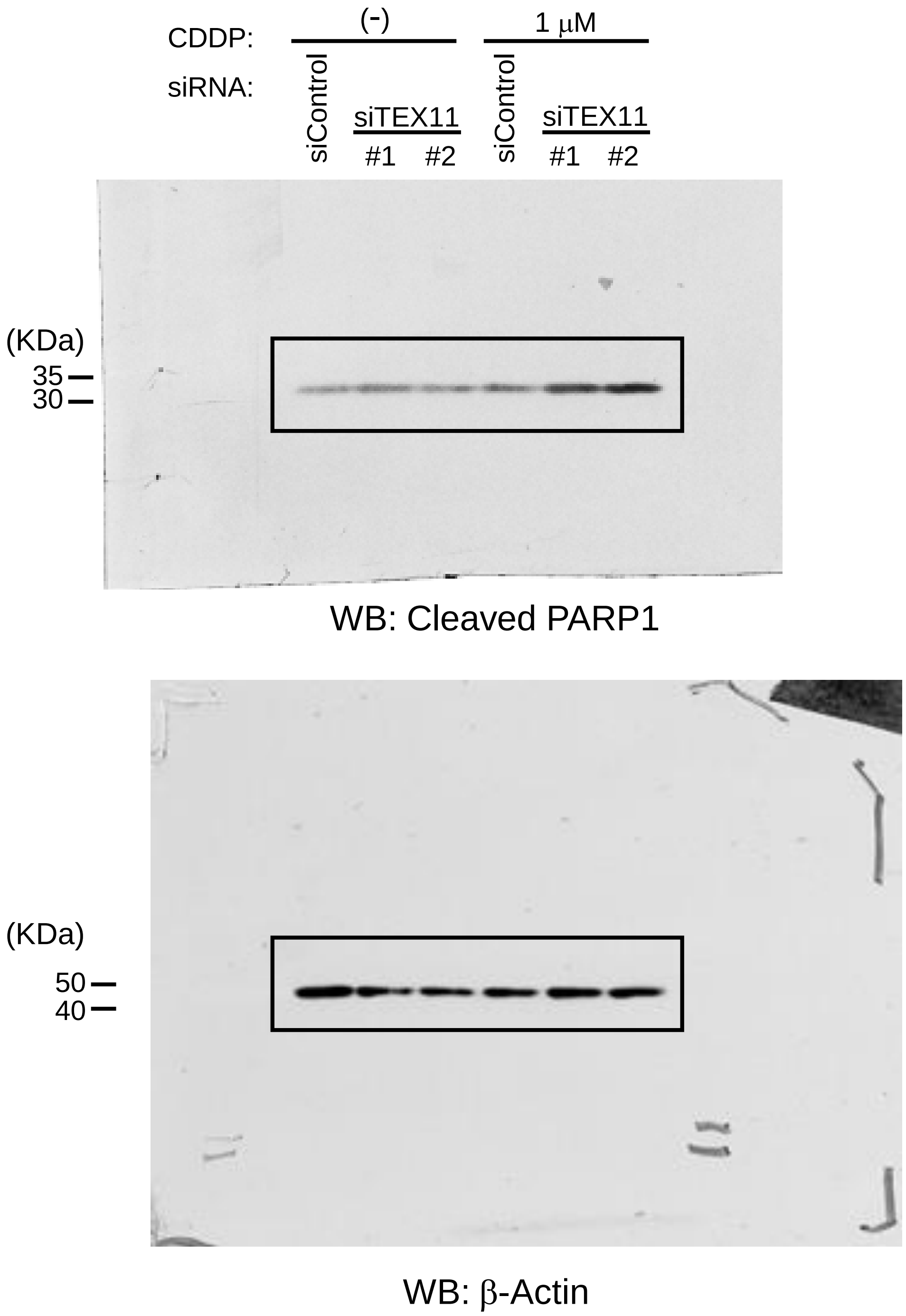

Supplementary Figure 6: Uncropped blot images in Figure 3a.

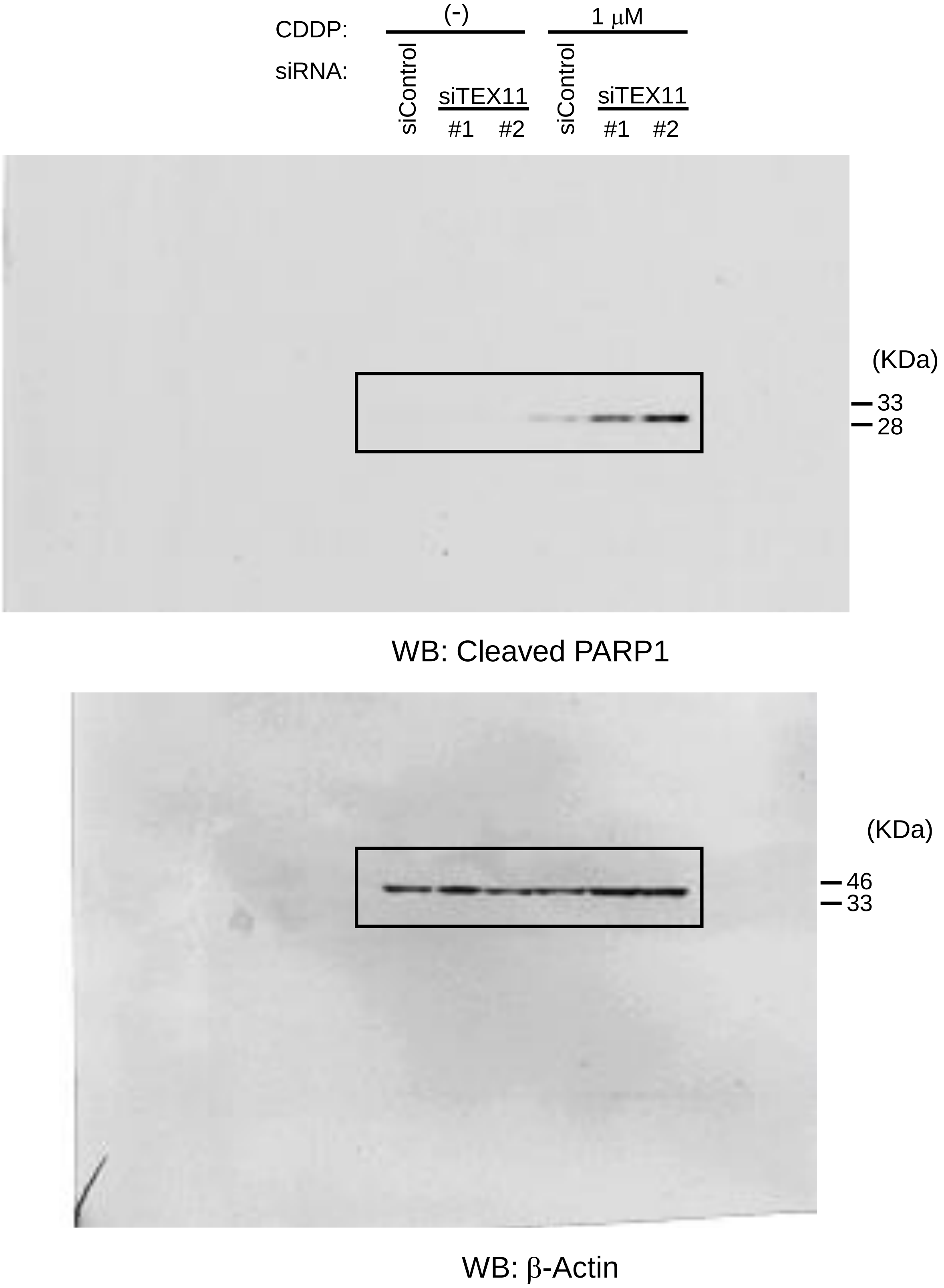

Supplementary Figure 7: Uncropped blot images in Figure 3b.

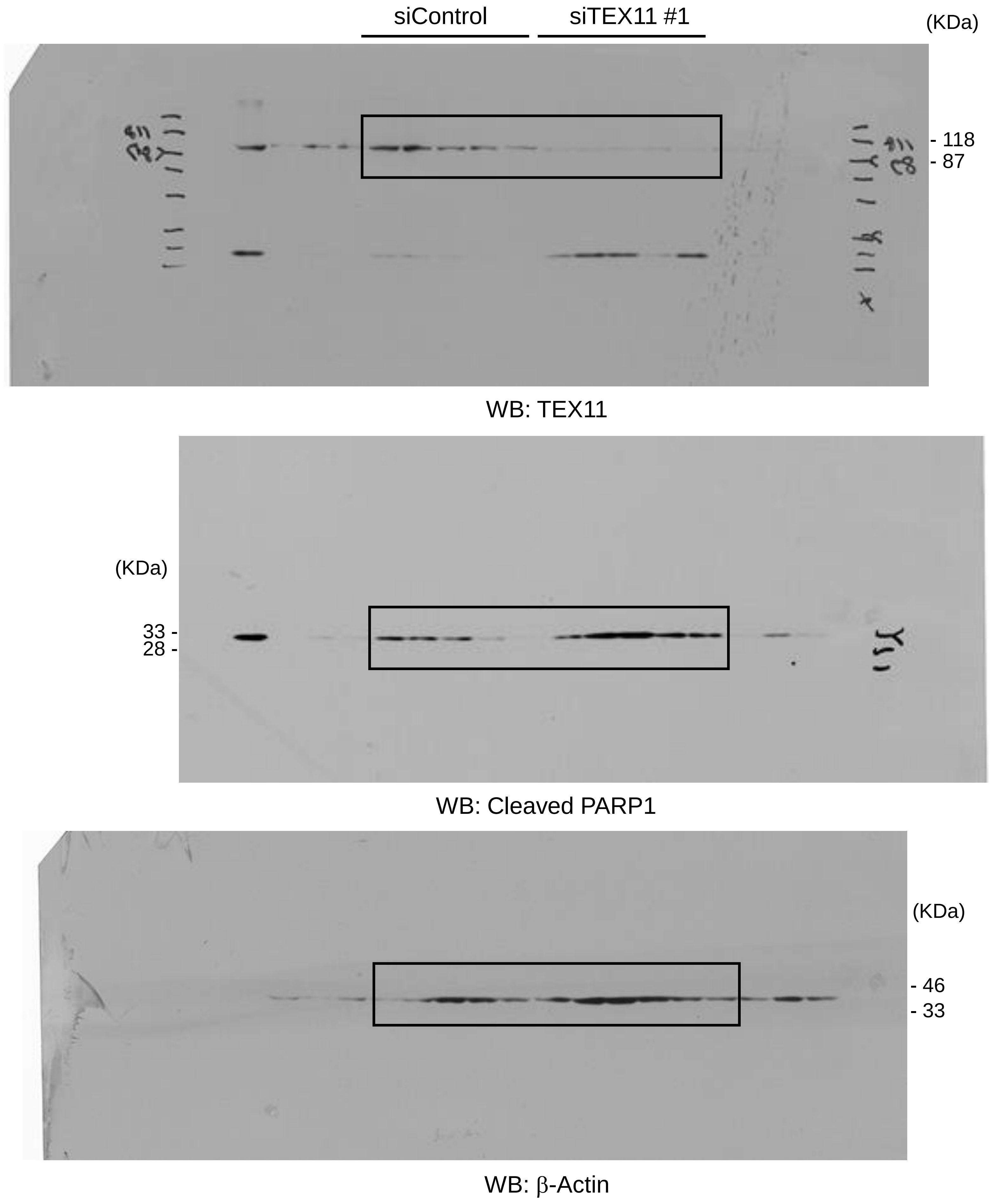

Supplementary Figure 8: Uncropped blot images in Figure 4f.

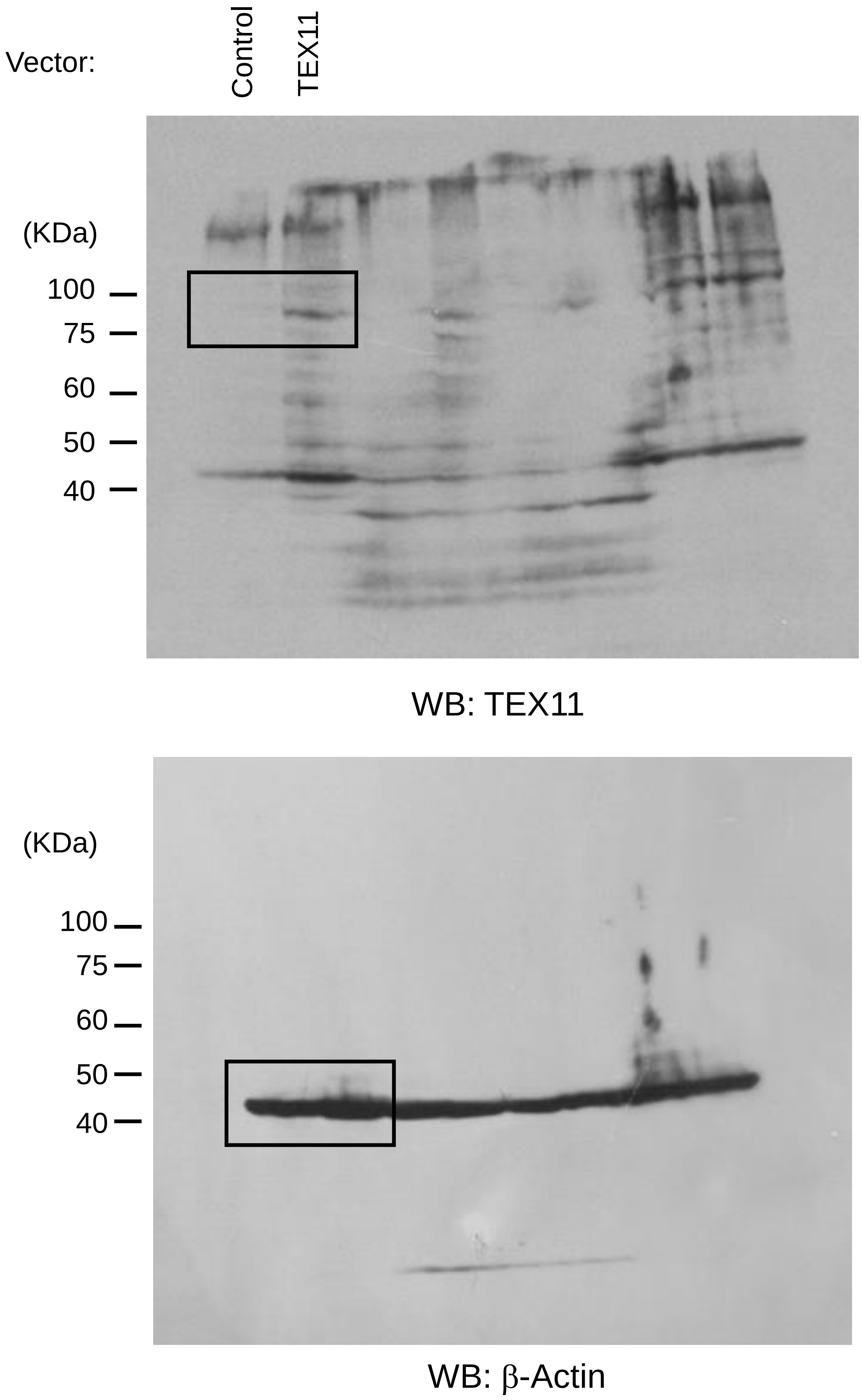

Supplementary Figure 9: Uncropped blot images in Supplementary Figure 4b.

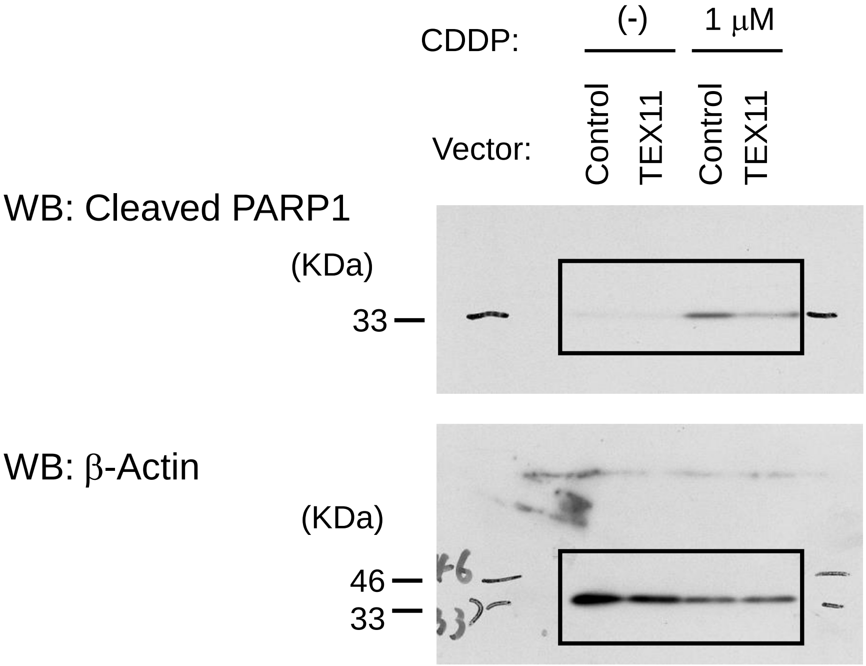

Supplementary Figure 10: Uncropped blot images in Supplementary Figure 4d.

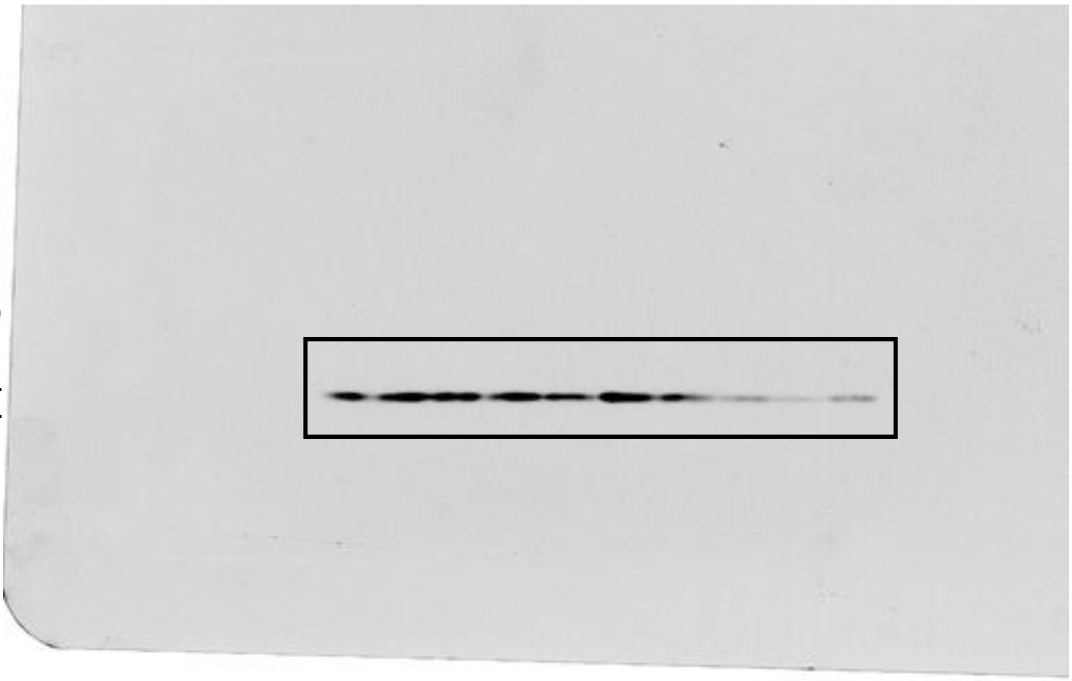

WB: PCNA

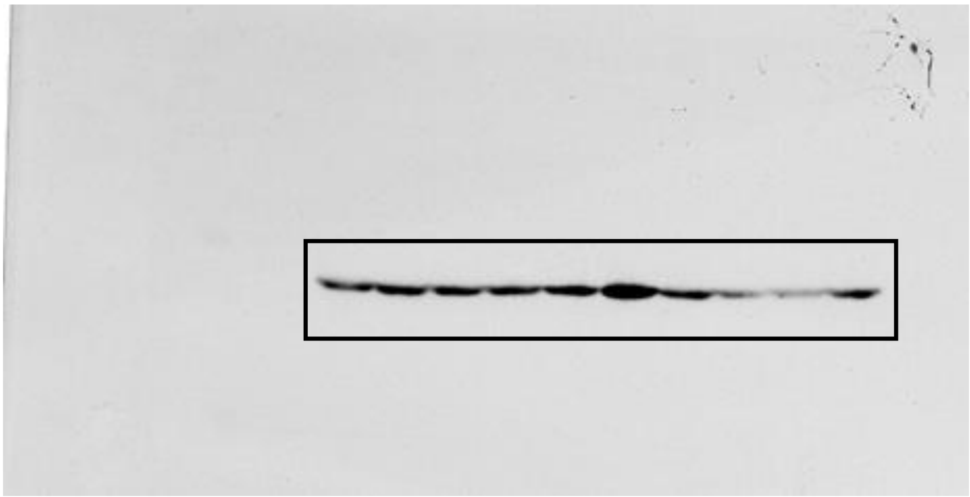

WB: β-Actin

Supplementary Figure 11: Uncropped blot images in Supplementary Figure 5a.
